# Mechanisms of Inhaled Fine Particulate Air Pollution–Induced Arterial Blood Pressure Changes

**DOI:** 10.1289/ehp.11573

**Published:** 2008-10-06

**Authors:** Carlo R. Bartoli, Gregory A. Wellenius, Edgar A. Diaz, Joy Lawrence, Brent A. Coull, Ichiro Akiyama, Lani M. Lee, Kazunori Okabe, Richard L. Verrier, John J. Godleski

**Affiliations:** 1 Molecular and Integrative Physiological Sciences Program, Department of Environmental Health, Harvard School of Public Health, Boston, Massachusetts, USA; 2 Department of Medicine, Beth Israel Deaconess Medical Center, Boston, Massachusetts, USA; 3 Department of Biostatistics, Harvard School of Public Health, Boston, Massachusetts, USA; 4 Sanyo National Hospital, Yamaguchi, Japan; 5 Department of Pathology, Brigham and Women’s Hospital, Boston, Massachusetts, USA

**Keywords:** α-adrenergic receptors, baroreceptors, blood pressure, hypertension, particulate air pollution

## Abstract

**Background:**

Epidemiologic studies suggest a positive association between fine particulate matter and arterial blood pressure, but the results have been inconsistent.

**Objectives:**

We investigated the effect of ambient particles on systemic hemodynamics during a 5-hr exposure to concentrated ambient air particles (CAPs) or filtered air (FA) in conscious canines.

**Methods:**

Thirteen dogs were repeatedly exposed via permanent tracheostomy to CAPs (358.1 ± 306.7 μg/m^3^, mean ± SD) or FA in a crossover protocol (55 CAPs days, 63 FA days). Femoral artery blood pressure was monitored continuously via implanted telemetry devices. We measured baroreceptor reflex sensitivity before and after exposure in a subset of these experiments (*n* = 10 dogs, 19 CAPs days, 20 FA days). In additional experiments, we administered α-adrenergic blockade before exposure (*n* = 8 dogs, 16 CAPs days, 15 FA days). Blood pressure, heart rate, rate–pressure product, and baroreceptor reflex sensitivity responses were compared using linear mixed-effects models.

**Results:**

CAPs exposure increased systolic blood pressure (2.7 ± 1.0 mmHg, *p* = 0.006), diastolic blood pressure (4.1 ± 0.8 mmHg; *p* < 0.001), mean arterial pressure (3.7 ± 0.8 mmHg; *p* < 0.001), heart rate (1.6 ± 0.5 bpm; *p* < 0.001), and rate–pressure product (539 ± 110 bpm × mmHg; *p* < 0.001), and decreased pulse pressure (−1.7 ± 0.7 mmHg, *p* = 0.02). These changes were accompanied by a 20 ± 6 msec/mmHg (*p* = 0.005) increase in baroreceptor reflex sensitivity after CAPs versus FA. After α-adrenergic blockade, responses to CAPs and FA no longer differed significantly.

**Conclusions:**

Controlled exposure to ambient particles elevates arterial blood pressure. Increased peripheral vascular resistance may mediate these changes, whereas increased baroreceptor reflex sensitivity may compensate for particle-induced alterations in blood pressure.

Epidemiologic studies have reported a consistent increased risk for cardiac morbidity and mortality associated with acute exposure to ambient particulate matter (PM) (Brook et al. 2004). The impact of ambient PM on arterial blood pressure may be an important factor in the observed adverse cardiovascular health effects. Several epidemiologic studies have reported associations between short-term fluctuations in ambient PM levels and arterial blood pressure. However, the direction of the observed changes has been inconsistent with studies reporting increases (Auchincloss et al. 2008; Ibald-Mulli et al. 2001; Linn et al. 1999; Urch et al. 2005; Zanobetti et al. 2004), decreases (Harrabi et al. 2006; Ibald-Mulli et al. 2004), or no associations (de Paula Santos et al. 2005; Jansen et al. 2005; Madsen and Nafstad 2006; Mar et al. 2005). The few animal toxicologic studies published to date have reported similarly discordant results and suggest that exposure to particles may either decrease (Cheng et al. 2003) or increase (Chang et al. 2004) blood pressure.

To investigate the effects of ambient PM on arterial blood pressure, we implanted telemetry blood pressure catheters and monitored femoral arterial blood pressure in canines during a 5-hr inhalation exposure to either concentrated ambient particles (CAPs) or filtered air (FA). To elucidate mechanisms involved in arterial blood pressure changes after inhalation of CAPs and the potential involvement of peripheral vaso-constriction in observed increases in arterial blood pressure, we determined baroreceptor reflex sensitivity before and after exposure to CAPs or FA and performed additional experiments with α-adrenergic blockade. The physiologic response mechanisms associated with exposure to ambient PM that we explored in this study have the potential to provide new insight into the acute health effects of inhaled ambient PM and to suggest explanations for the contradictory findings in previous studies.

## Materials and Methods

### Surgical preparation

We treated animals humanely and with regard for alleviation of suffering using protocols approved by the Harvard Medical Area Standing Committee on Animals. Thirteen female mixed-breed dogs obtained from either Butler Farms (Clyde, NY, USA) or Marshall Laboratories (North Rose, NY, USA), 2–12 years of age (11 animals ~ 2–3 years of age, 2 animals ~ 12 years of age) and weighing an average of 15.7 kg (range, 13.6–18.2 kg) were used for this study.

We performed all surgeries aseptically while animals were maintained under a surgical level of anesthesia. Animals were preanesthetized using ketamine (10 mg/kg bolus), xylazine (1.5 mg/kg bolus), and atropine (0.04 mg/kg bolus). An endotracheal tube was inserted to initiate mechanical ventilation with 1–3% isoflurane and pure oxygen for anesthesia. Telemeter units (D70-CCTP; Data Sciences International, St. Paul, MN, USA) were implanted in the left flank. The blood pressure catheter was tunneled subcutaneously to the inguinal region and implanted 4–6 cm, oriented upstream in the femoral artery using a technique similar to our previously described method (Bartoli et al. 2006). We created a permanent tracheostomy to facilitate exposure, as previously described (Bartoli et al. 2008; Godleski et al. 2000). Breathing via tracheostomy has minimal effects on airway mechanics (Drazen et al. 1982). After surgery, animals recovered for a minimum of 3 weeks. Animals were acclimatized to the laboratory, exposure chamber, and all aspects of the experimental protocol before beginning experimentation.

After sacrifice and histopathologic examination, it was determined that during the course of the protocol, one animal developed an approximately 1 cm × 1 cm anterior left ventricle myocardial infarction.

### Exposure technology and characterization

The characteristics of the Harvard Ambient Particle Concentrator (HAPC) and exposure chamber are well documented (Godleski et al. 2000; Sioutas et al. 1995). The HAPC concentrates ambient fine PM with an aerodynamic diameter between 0.15 and 2.5 μm to approximately 30 times ambient levels with minimal effects on the particle size distribution or chemical composition. Particles with diameters > 2.5 μm are removed upstream of the HAPC, and particles with diameters < 0.15 μm and ambient gases are neither enriched nor excluded. We mounted the ambient outdoor intake of the HAPC through the window of the exposure laboratory located on the first floor and positioned it approximately 1 m from the building and 100 m from the nearest roadway.

Exposures typically took place between 0800 hours and 1430 hours each day. We measured CAPs mass concentration continuously using a tapered-element oscillating microbalance (TEOM Series 1400a; Rupprecht & Patashnick Co., Inc., East Greenbush, NY, USA), black carbon concentration by optical transmission (aethalometer model AE-9; Magee Scientific, Berkeley, CA, USA), and CAPs particle number concentration using a condensation particle counter (CPC model 3022A; TSI, Inc., Shoreview, MN, USA), as previously described (Godleski et al. 2000).

### Experimental design and data acquisition

To evaluate the acute effects of ambient PM on arterial blood pressure, animals were repeatedly exposed for 5 hr to either CAPs or FA in pairs in a crossover protocol. Within each pair, one subject was assigned to CAPs exposure and the other was assigned to FA exposure. We separated exposure days by a week or more, during which no exposures took place. Blood pressure data were available from 13 animals exposed to FA on 63 days and CAPs on 55 days (Table 1).

Baroreceptor reflex sensitivity was assessed in 10 of these dogs during 20 exposures to FA and 19 exposures to CAPs. We assessed baroreceptor reflex sensitivity within 20 min before the start of exposure and within 20 min after the end of exposure using the method of Smyth et al. (1969). Briefly, the sensitivity of the baroreceptor reflex to transient hypertension was assessed by determining the slope of the regression line relating the prolongation of the R-R interval to the rise in systolic arterial pressure during a transient elevation of arterial pressure induced by an intravenous injection of phenylephrine (10 μg/kg).

We conducted additional experiments with prazosin, an α-adrenergic antagonist, in 8 of the original 13 animals (*n* = 15 FA exposures, 16 CAPs exposures). In these experiments, oral prazosin (0.065 mg/kg) was administered 30–60 min before exposure to induce α-adrenergic blockade during exposures. Immediately after each exposure, phenylephrine (10 μg/kg) was injected intravenously to verify continued α-adrenergic blockade. Use of dogs in a particular study was based on availability and operative hardware.

Continuous arterial blood pressure was monitored and recorded throughout exposures (DSI Dataquest ART 3.1; Data Sciences International). We derived systolic, diastolic, mean, and pulse arterial pressures and heart rate from the arterial blood pressure signal. Rate–pressure product, a standard index of myocardial metabolic demand, was calculated as the product of heart rate and systolic blood pressure (Rooke and Feigl 1982). Data were exported and analyzed with custom-designed Matlab software (Mathworks, Inc., Natick, MA, USA) to obtain 5-min averages for each outcome.

### Statistical analysis

Descriptive statistics for each dog were evaluated for exposure measures (CAPs mass and number concentration, black carbon concentration) and cardiovascular parameters (systolic, diastolic, mean, and pulse arterial pressure, heart rate, and rate–pressure product). We applied mixed-effects models to the 5-min averages for each cardiovascular parameter. In a first analysis applied to non-prazosin and prazosin exposures separately, we used a model containing CAPs as a binary variable to assess overall differences between CAPs and FA responses. In addition, to account for dog heterogeneity as well as day-to-day variability, the models contained dog and date as random effects. Thus, the model estimated within-day CAPs versus FA differences by comparing an individual dog’s average response to that of its chambermate after adjusting for each animal’s overall average response. The models also contained first-order autoregressive serially correlated errors to account for time series correlation within each dog exposure. This modeling strategy generally minimized the Akaike information criterion (Fitzmaurice et al. 2004) for most parameters. Robustness of the resulting inferences were verified by considering a second model that used empirical standard errors known to be less sensitive to the chosen covariance structure of the repeated measures (Venables and Ripley 1994), as well as a third model that relaxed the first-order auto regressive assumption with a less restrictive banded Toeplitz structure of order 10. We assessed differences between CAPs effects during nonprazosin versus prazosin exposure by applying the same model, but with a prazosin main effect and with exposure × prazosin interaction terms added. The effect of CAPs exposure on baroreceptor reflex sensitivity was assessed using a linear mixed-effects model with fixed effects for exposure type and a random intercept for each dog.

In a second analysis, we assessed dose–response relationships using univariate analyses in which we fit the above repeated-measures regression model to each of the continuous measures, substituting the CAPs binary term with CAPs mass concentration, particle count, or black carbon integrated over the 5-hr exposure period.

Mixed model analyses were performed using PROC MIXED in SAS version 9 (SAS Institute Inc., Cary, NC, USA). All statistical tests are two-tailed, and *p*-values < 0.05 were considered statistically significant.

## Results

To evaluate the effect of CAPs exposure on systemic hemodynamics, we repeatedly exposed 13 animals to either CAPs (*n* = 55 exposure days) or FA (*n* = 63 exposure days) in a crossover protocol. Linear mixed-effects models accounted for the unbalanced crossover experimental design as well as the correlation among the multiple measurements made within each animal. Animals did not exhibit observable behavioral differences during exposure to CAPs versus exposure to FA.

**Figure 1:**
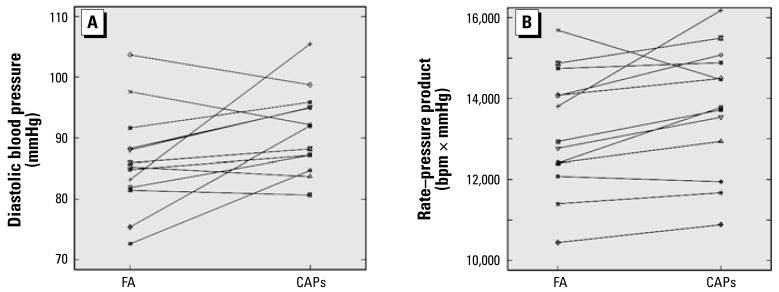
Average diastolic blood pressure (*A*) or rate–pressure product (*B*) by dog during exposure to either FA or CAPs. Each point represents the crude mean response across all FA or CAPs exposures for a single dog. Responses from the same dog are connected by a solid line. Table 1 shows the numbers of exposures for each dog.

CAPs exposure was associated with statistically significant increases in systolic blood pressure, diastolic blood pressure, and mean arterial pressure compared with FA exposure (Table 2). We observed the largest absolute effect for diastolic blood pressure, which increased by an average of 4.1 ± 0.8 mmHg (*p* < 0.001). CAPs exposure was also associated with a statistically significant increase in heart rate and rate–pressure product (Table 2). Figure 1 shows individual dog responses for diastolic blood pressure and rate–pressure product. CAPs exposure was associated with increased diastolic blood pressure in 9 of the 13 animals and with increased rate–pressure product in 10 of the 13 animals. Results from the two elderly animals were within the range of observed individual effects of the 11 younger animals.

**Figure 2:**
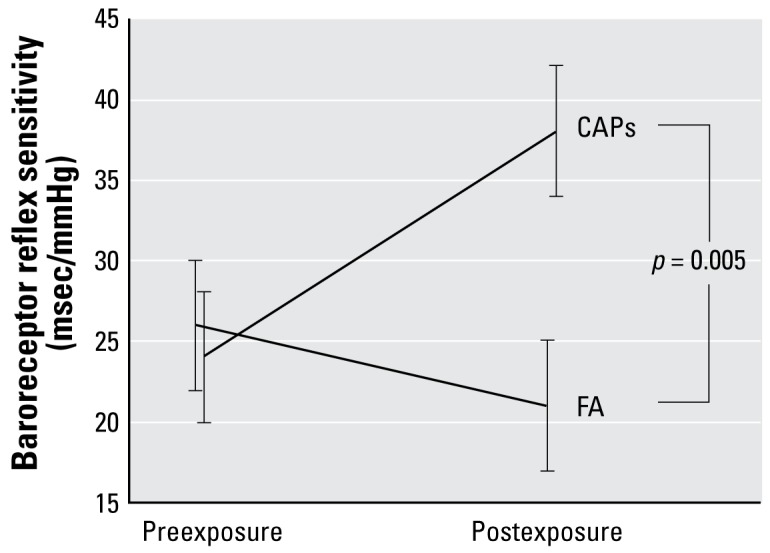
Baroreceptor reflex sensitivity before and after 5-hr inhalation exposures to CAPs or FA in 10 dogs. The change in baroreceptor reflex sensitivity was significantly different after CAPs versus FA exposures.

In 10 of these dogs undergoing 20 FA exposures and 19 CAPs exposures, we assessed the effects of CAPs exposure on baroreceptor reflex function (Figure 2). Overall, CAPs exposure significantly increased baroreceptor reflex sensitivity by 20 ± 6 msec/mmHg compared with FA (*p* = 0.005).

To evaluate the role of α-adrenergic receptors in the CAPs-induced pressure changes, we conducted additional experiments with prazosin, an α-adrenergic antagonist, in 8 of the original 13 animals (*n* = 15 FA exposures, 16 CAPs exposures). As expected, α-adrenergic blockade decreased average levels of systolic and diastolic blood pressure, mean arterial pressure, pulse pressure, and rate–pressure product. After prazosin administration, CAPs exposure was not associated with statistically significant changes in any hemodynamic parameter (Table 3), but we had limited statistical power to detect effects of this magnitude in this sample. However, we observed significant CAPs-by-prazosin interactions for pulse pressure, heart rate, and rate–pressure product (*p* < 0.02 for each), suggesting that α-adrenergic blockade modified the response to CAPs for these parameters. The CAPs-by-prazosin interaction was marginally significant (*p* = 0.062) for diastolic blood pressure and not significant for systolic blood pressure (*p* = 0.99).

### CAPs characteristics

**Figure 3:**
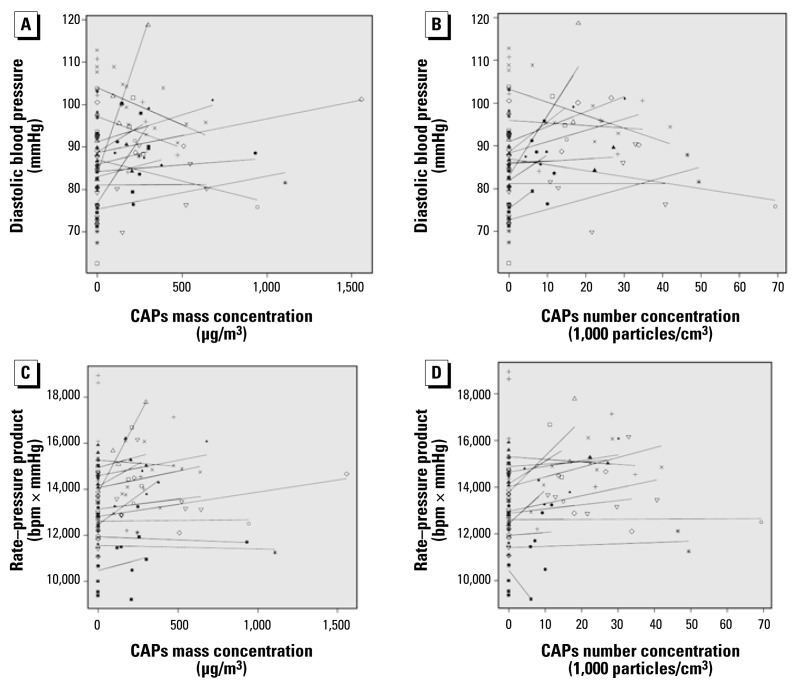
Estimated dose–response relationships by dog for diastolic blood pressure (*A* and *B*) and rate–pressure product (*C* and *D*). Each point represents the average of all measurements across a single 5-hr exposure period in a single dog. Each symbol represents data from a different dog, and each solid line represents the dog-specific slope and intercept from a linear model relating the given exposure metric to the given outcome.

Daily CAPs fine mass concentration ranged from 94.1 to 1557.0 μg/m^3^ (358.1 ± 306.7 μg/m^3^, mean ± SD), black carbon concentrations ranged from 1.3 to 32.0 μg/m^3^ (7.5 ± 6.1 μg/m^3^), and particle count concentrations ranged from 3,000 to 69,300 particles/cm^3^ (18,230 ± 13.151 particles/cm^3^). When included in the model as a covariate, CAPs mass, black carbon, and particle number concentrations were positively and significantly associated with each of the measured cardiovascular parameters except for pulse pressure (Table 4). Figure 3 shows estimated dose–response relationships by dog for diastolic blood pressure and rate–pressure product. These associations were no longer apparent in the subset of experiments in which animals received prazosin.

## Discussion

Our results suggest that acute inhalation exposure to CAPs increases arterial blood pressure in chronically instrumented canines. Changes in baroreceptor reflex sensitivity may have counteracted these changes, which are likely mediated by increased peripheral vascular resistance. Specifically, we observed that *a*) CAPs exposure increased systolic blood pressure, diastolic blood pressure, mean arterial pressure, heart rate, and rate–pressure product, and decreased pulse pressure; *b*) CAPs exposure significantly increased baroreceptor reflex sensitivity; and *c* ) CAPs-related changes in pulse pressure, heart rate, and rate–pressure product were attenuated by the α-adrenergic antagonist prazosin.

The finding that CAPs exposure was associated with increased arterial blood pressure changes is consistent with most (Auchincloss et al. 2008; Ibald-Mulli et al. 2001; Linn et al. 1999; Urch et al. 2005; Zanobetti et al. 2004) but not all (Harrabi et al. 2006; Ibald-Mulli et al. 2001, 2004; Linn et al. 1999; Urch et al. 2005; Zanobetti et al. 2004) human epidemiologic studies. The studies that found a positive association report magnitudes of arterial blood pressure changes similar to those we found in the present study. Moreover, the statistically significant increases in diastolic blood pressure Urch et al. (2005) observed in healthy adults after 2-hr laboratory exposure to CAPs and ozone are consistent with the short-latency effects seen in the present study.

We are aware of only two previous toxicologic studies evaluating the effect of ambient PM on arterial blood pressure. Cheng et al. (2003) studied three monocrotaline-treated rats and found that CAPs exposure was associated with significant reductions in mean arterial pressure and heart rate at 1–2 hr after the start of exposure. However, it is unclear if the investigators measured systemic or pulmonary arterial pressure. Furthermore, in their model of pulmonary hypertension and right ventricular hypertrophy, a decreased systemic arterial pressure is likely indicative of right heart failure. Subsequently, the same group studied four spontaneously hypertensive rats and found that CAPs exposure significantly increased mean arterial pressure in the spring but had no significant effect in the summer (Chang et al. 2004).

Several physiologic mechanisms may account for our findings. Changes in peripheral vascular resistance—mediated by neurohormonal activation or local metabolic factors such as nitric oxide (NO)—are important for acute blood pressure regulation. In the peripheral circulation, norepinephrine released from sympathetic nerves and circulating nor-epinephrine released from the adrenal medulla binds to α_1_ adrenergic receptors and induces vasoconstriction. To test whether the CAPs-induced increases in blood pressure were mediated by neurohormonal activation, we pretreated some animals with prazosin, a selective α_1_ adrenergic antagonist with minimal effects on cardiac function (Graham, 1984). We found that CAPs exposure was associated with smaller, nonsignificant increases in arterial blood pressure in the presence of α-adrenergic blockade compared with untreated animals, suggesting that the observed effects of CAPs were due at least in part to α-adrenergic receptor activation and increases in peripheral vascular resistance. Our finding in untreated animals that CAPs exposure decreased pulse pressure in the context of preferential increases in diastolic blood pressure further supports the role of increased peripheral vascular resistance.

The above findings are consistent with the large body of toxicologic and epidemiologic literature indicating that exposure to ambient PM is associated with increased sympathetic nervous system activity. With that in mind, as well as the documented inverse relationship between circulating levels of catecholamines and baroreceptor reflex sensitivity (Goldstein 1983), our *a priori* hypothesis was that CAPs exposure would decrease the baroreflex response. Contrary to expectation, we found that CAPs exposure increased baroreceptor reflex sensitivity compared with FA. An increase in baroreceptor reflex sensitivity is consistent with an up-regulation of vagal reflexes (Schwartz et al. 1988). In previous experiments in this animal model and using measures of heart rate variability, we have shown that CAPs exposure leads to increases in both sympathetic and parasympathetic autonomic tone, although relative parasympatho-excitation predominates (Godleski et al. 2000). Therefore, air pollution–mediated hemodynamic changes may be attributable to phasic or tonic up-regulation of both sympathetic and parasympathetic tone, which may offset each other and account for the minimal change to heart rate that was observed. Alternatively, the increased baroreceptor reflex sensitivity that we observed may represent activation of a compensatory mechanism to restore arterial blood pressure toward pre-exposure levels.

Alterations in endothelial function and subsequent changes in vasomotor tone may have also played a role in the observed responses to CAPs. Indeed, plasma markers of endothelial cell activation, including von Willebrand factor, endothelin-1, and soluble intracellular adhesion molecule-1 (sICAM-1), have been associated with short-term exposure to PM in animals and/or humans (Ando et al. 2001; Barregard et al. 2006; Bouthillier et al. 1998; Liao et al. 2005; Peretz et al. 2008). Also, reduced brachial artery diameter in response to a 2-hr exposure to CAPs and ozone (Brook et al. 2002) and decreased brachial artery reactivity associated with ambient PM (O’Neill et al. 2005) have been reported in humans.

Finally, the observed results may also have been due to alterations in cardiac output, defined as the product of heart rate and stroke volume. In the present study, CAPs exposure was associated with increased heart rate and rate–pressure product, an index of cardiac metabolic demand, suggesting that changes in cardiac output may have also played a role in the observed responses. Studies with measures of cardiac output are needed to explore this hypothesis further.

The concentration and composition of ambient air pollution exhibited daily, weekly, and seasonal variability and were presumably influenced by traffic patterns as well as wind and weather patterns. The process of concentrating the particles increases these known variations more than 30-fold and allows the assessment of specific dose–response relationships. Because these experiments were typically performed between 0800 hours and 1430 hours each day and data were collected during all four seasons, the observed CAPs fine mass variability, was expected and was similar to values previously reported by our group (Godleski et al. 2000; Wellenius et al. 2003). A strength of this experimental approach is that we exposed animals to elevated concentrations of real-world particles, so the results of our experiments may be interpreted as the response of dogs exposed to the same mix of particles to which Boston area residents are exposed on those days.

Several studies suggest that traffic-related pollution estimated by black carbon concentration or ultrafine PM concentration estimated by particle counts might be responsible for the observed associations between ambient PM levels and cardiovascular health effects. Likewise, we found an association between CAPs mass, black carbon, and particle count concentrations and all of the cardiovascular parameters except for pulse pressure. These results warrant further investigation of specific sources or constituents of ambient PM responsible for cardiovascular changes. Additionally, the effects of ambient PM on hemodynamics in other vascular beds, including the coronary circulation, are currently unknown and merit further investigation.

Although the absolute magnitude of the average change in blood pressure in response to CAPs may appear small (on the order of 2–4 mmHg), such an increase applied over time to a large population could have important public health consequences, as documented by results from multiple large prospective cohorts (Kannel et al. 2003). Moreover, the average response necessarily obscures the underlying heterogeneity between the animals. As shown in Figure 1, the hemodynamic response to CAPs exhibited considerable dog-to-dog variability. This heterogeneity may suggest that certain animals were more susceptible to the effects of CAPs. For example, the one dog that developed an anterior left ventricle infarct presented some of the largest increases in arterial blood pressure observed during the course of the study. Determining genetic or phenotypic correlates of susceptibility in humans and animals remains an important direction for future research. Whether pharmacologic intervention may attenuate air pollution–mediated hypertensive responses in humans, as it does in dogs, is unknown.

### Strengths and limitations

The present study has several potential limitations. First, we did not directly measure cardiac output, total peripheral vascular resistance, or plasma levels of catecholamines. Thus, we based our conclusions regarding the effects of CAPs on these parameters on inferences, which need to be confirmed or refuted in subsequent experiments. Second, we did not continue hemodynamic recordings after the end of exposures, and thus, we do not know the duration of the observed effects. Third, upper-airway receptors in the nostrils, nasopharynx, and trachea may participate in respiratory and cardiovascular responses. In our canine model, inhalation exposure to CAPs occurs via a permanent tracheostomy (Bartoli et al. 2008), which bypasses the nasopharynx and may exclude important pathophysiologic pathways, especially in canines, which rely heavily on olfactory senses. Fourth, we performed this study in healthy, female dogs. Although canines are an excellent cardiovascular model species for humans, species differences remain that limit direct extrapolation of these results to human populations. Moreover, whether the observed results are influenced by sex, age, or preexisting cardiovascular disease is unknown, although anecdotally, the one animal with a myocardial infarction exhibited some of the largest increases in arterial blood pressure. Notwithstanding these limitations, the present study has several strengths, including the use of a large animal model, a longitudinal repeated-measures study design, continuous high-fidelity blood pressure recordings, and inhalation exposure to real-world ambient PM at realistic concentrations.

The results of the present study suggest that statistically and clinically significant increases in diastolic blood pressure are associated with exposure to ambient urban PM, implicate peripheral vascular α-adrenergic receptors in the response, and provide evidence for a physiologic compensatory response. Clearly, additional studies are needed to fully elucidate the mechanisms underlying the observed effects and to determine the sources or constituents of ambient PM responsible for these effects. In particular, the role of β-adrenergic receptors and vasoactive compounds in these hemodynamic responses remains uncertain.

## Figures and Tables

**Table 1:** Number of exposure days by dog, exposure FA or CAPs, and experiment.

	Untreated		BRS		Prazosin	
Dog	FA	CAPs	FA	CAPs	FA	CAPs
Dog 1	4	3	2	2	2	2
Dog 2	3	2				
Dog 3^a^	5	3	2	2	2	3
Dog 4	6	6	3	2		
Dog 5	6	11	1	2	3	2
Dog 6	8	4	3	1		
Dog 7	8	7	2	3	2	3
Dog 8	3	2				
Dog 9	4	4	3	2	2	3
Dog 10	3	5	2	2	1	0
Dog 11	5	3	1	2	2	2
Dog 12	3	2				
Dog 13	5	3	1	1	1	1
Total exposure days	63	55	20	19	15	16

BRS, baroreceptor reflex sensitivity.

a This animal developed an anterior left ventricular myocardial infarction during the course of the study.

**Table 2:** Hemodynamic responses (mean ± SD) during 5-hr exposure to FA or CAPs in 13 dogs.

Parameter	FA^a^ (*n* = 63 days)	CAPs *a* (*n* = 53 days)	Crude difference^b^	Adjusted difference^c^	*p*-Value^c^
Systolic pressure (mmHg)	138.3 ± 19.9	140.9 ± 19.3	2.6	2.7 ± 1.0	0.006
Diastolic pressure (mmHg)	87.2 ± 13.8	91.9 ± 12.1	4.7	4.1 ± 0.8	< 0.001
Mean pressure (mmHg)	104.3 ± 14.6	108.2 ± 12.8	3.9	3.7 ± 0.8	< 0.001
Pulse pressure (mmHg)	51.1 ± 14.6	49.0 ± 16.2	−2.1	−1.7 ± 0.7	0.02
Heart rate (bpm)	98.0 ± 13.7	98.4 ± 11.6	0.4	1.6 ± 0.5	< 0.001
Rate–pressure product (bpm × mmHg)	13,426 ± 2,465	13,821 ± 2,337	395	539 ± 110	< 0.001

a Mean ± SD from 5- min average data.

b Estimated as difference between mean levels during FA and CAPs exposures.

c Estimated effect of CAPs exposure ± SE from a linear mixed model controlling for dog as a fixed effect, week-within-dog as a random effect, and first-order autoregressive serially correlated errors over time within exposure group.

**Table 3:** Hemodynamic responses (mean ± SD) during 5-hr exposure to FA or CAPs in eight dogs treated with prazosin, an α-adrenergic antagonist.

Parameter	FA^a^ (*n* = 15 days)	CAPs^a^ (*n* = 16 days)	Crude difference^b^	Adjusted difference^c^	*p*-Value^c^
Systolic pressure (mmHg)	119.8 ± 22.8	119.1 ± 20.3	−0.7	2.8 ± 2.8	0.33
Diastolic pressure (mmHg)	81.9 ± 18.1	79.6 ± 14.8	−2.3	1.3 ± 1.5	0.40
Mean pressure (mmHg)	94.4 ± 18.4	92.8 ± 15.2	−1.6	1.8 ± 1.9	0.34
Pulse pressure (mmHg)	38.0 ± 15.4	39.5 ± 15.5	1.5	1.4 ± 1.2	0.26
Heart rate (bpm)	98.4 ± 13.4	96.9 ± 10.8	−1.5	−1.8 ± 1.0	0.06
Rate–pressure product (bpm × mmHg)	11,779 ± 2,686	11,548 ± 2,361	−231	8 ± 198	0.97

a Mean ± SD of 5-min average data.

b Estimated as difference between mean levels during FA and CAPs exposures.

c Estimated effect of CAPs exposure ± SE from a linear mixed model controlling for dog as a fixed effect, week-within -dog as a random effect, and first-order autoregressive serially correlated errors over time within exposure group.

**Table 4:** Estimated hemodynamic response for an interquartile range increase in fine PM mass, black carbon, and particle count concentrations.

	Fine PM mass			Black carbon			Particle count		
Parameter	Effect	95% CI	*p*-Value	Effect	95% CI	*p*-Value	Effect	95% CI	*p*-Value
Systolic pressure (mmHg)	1.1	0.0 to 2.1	0.050	1.3	−0.1 to 2.7	0.074	2.6	0.8 to 4.4	0.004
Diastolic pressure (mmHg)	1.1	0.2 to 2.1	0.02	1.9	0.7 to 3.1	0.002	1.8	0.3 to 3.3	0.017
Mean pressure (mmHg)	1.1	0.2 to 2.0	0.02	1.6	0.4 to 2.8	0.007	2.0	0.5 to 3.5	0.008
Pulse pressure (mmHg)	−0.4	−1.3 to 0.5	0.40	−0.9	−2.1 to 0.2	0.11	0.3	−0.9 to 1.5	0.63
Heart rate (bpm)	0.9	0.3 to 1.5	0.004	1.8	1.0 to 2.5	< 0.001	2.4	1.5 to 3.3	< 0.001
Rate–pressure product (bpm × mmHg)	212	84 to 341	0.001	351	185 to 517	< 0.001	617	415 to 819	< 0.001

Interquartile ranges were 231.6 μg/m^3^ for mass concentration, 19,650 particles/cm^3^ for particle number concentration, and 6.5 μg/m^3^ for black carbon concentration.

## References

[b1-ehp-117-361] Ando M, Shima M, Adachi M, Tsunetoshi Y (2001). The role of intercellular adhesion molecule-1 (ICAM-1), vascular cell adhesion molecule-1 (VCAM-1), and regulated on activation, normal T-cell expressed and secreted (RANTES) in the relationship between air pollution and asthma among children. Arch Environ Health.

[b2-ehp-117-361] Auchincloss AH, Roux AV, Dvonch JT, Brown PL, Barr RG, Daviglus ML (2008). Associations between recent exposure to ambient fine particulate matter and blood pressure in the multi-ethnic study of atherosclerosis (MESA). Environ Health Perspect.

[b3-ehp-117-361] Barregard L, Sallsten G, Gustafson P, Andersson L, Johansson L, Basu S (2006). Experimental exposure to wood-smoke particles in healthy humans: effects on markers of inflammation, coagulation, and lipid peroxidation. Inhal Toxicol.

[b4-ehp-117-361] Bartoli CR, Akiyama I, Okabe K, Diaz EA, Godleski JJ (2008). Permanent tracheostomy for long-term respiratory studies. J Surg Res.

[b5-ehp-117-361] Bartoli CR, Okabe K, Akiyama I, Verrier RL, Godleski JJ (2006). Technique for implantation of chronic indwelling aortic access catheters. J Invest Surg.

[b6-ehp-117-361] Bouthillier L, Vincent R, Goegan P, Adamson IY, Bjarnason S, Stewart M (1998). Acute effects of inhaled urban particles and ozone: lung morphology, macrophage activity, and plasma endothelin-1. Am J Pathol.

[b7-ehp-117-361] Brook RD, Brook JR, Urch B, Vincent R, Rajagopalan S, Silverman F (2002). Inhalation of fine particulate air pollution and ozone causes acute arterial vasoconstriction in healthy adults. Circulation.

[b8-ehp-117-361] Brook RD, Franklin B, Cascio W, Hong Y, Howard G, Lipsett M (2004). Air pollution and cardiovascular disease: a statement for healthcare professionals from the Expert Panel on Population and Prevention Science of the American Heart Association. Circulation.

[b9-ehp-117-361] Chang CC, Hwang JS, Chan CC, Wang PY, Hu TH, Cheng TJ (2004). Effects of concentrated ambient particles on heart rate, blood pressure, and cardiac contractility in spontaneously hypertensive rats. Inhal Toxicol.

[b10-ehp-117-361] Cheng TJ, Hwang JS, Wang PY, Tsai CF, Chen CY, Lin SH (2003). Effects of concentrated ambient particles on heart rate and blood pressure in pulmonary hypertensive rats. Environ Health Perspect.

[b11-ehp-117-361] de Paula Santos U, Braga AL, Giorgi DM, Pereira LA, Grupi CJ, Lin CA (2005). Effects of air pollution on blood pressure and heart rate variability: a panel study of vehicular traffic controllers in the city of Sao Paulo, Brazil. Eur Heart J.

[b12-ehp-117-361] Drazen JM, O’Cain CF, Ingram RH (1982). Experimental induction of chronic bronchitis in dogs: effects on airway obstruction and responsiveness. Am Rev Respir Dis.

[b13-ehp-117-361] Fitzmaurice GM, Laird NM, Ware JH (2004). Applied Longitudinal Analysis.

[b14-ehp-117-361] Godleski JJ, Verrier RL, Koutrakis P, Catalano P, Coull B, Reinisch U (2000). Mechanisms of morbidity and mortality from exposure to ambient air particles. Res Rep Health Eff Inst.

[b15-ehp-117-361] Goldstein DS (1983). Arterial baroreflex sensitivity, plasma cat-echolamines, and pressor responsiveness in essential hypertension. Circulation.

[b16-ehp-117-361] Graham RM (1984). Selective alpha1-adrenergic antagonists: therapeutically relevant antihypertensive agents. Am J Cardiol.

[b17-ehp-117-361] Harrabi I, Rondeau V, Dartigues JF, Tessier JF, Filleul L (2006). Effects of particulate air pollution on systolic blood pressure: a population-based approach. Environ Res.

[b18-ehp-117-361] Ibald-Mulli A, Stieber J, Wichmann HE, Koenig W, Peters A (2001). Effects of air pollution on blood pressure: a population-based approach. Am J Public Health.

[b19-ehp-117-361] Ibald-Mulli A, Timonen KL, Peters A, Heinrich J, Wolke G, Lanki T (2004). Effects of particulate air pollution on blood pressure and heart rate in subjects with cardiovascular disease: a multicenter approach. Environ Health Perspect.

[b20-ehp-117-361] Jansen KL, Larson TV, Koenig JQ, Mar TF, Fields C, Stewart J (2005). Associations between health effects and particulate matter and black carbon in subjects with respiratory disease. Environ Health Perspect.

[b21-ehp-117-361] Kannel WB, Vasan RS, Levy D (2003). Is the relation of systolic blood pressure to risk of cardiovascular disease continuous and graded, or are there critical values?. Hypertension.

[b22-ehp-117-361] Liao D, Heiss G, Chinchilli VM, Duan Y, Folsom AR, Lin HM (2005). Association of criteria pollutants with plasma hemostatic/inflammatory markers: a population-based study. J Expo Anal Environ Epidemiol.

[b23-ehp-117-361] Linn WS, Gong H, Clark KW, Anderson KR (1999). Day-to- day particulate exposures and health changes in Los Angeles area residents with severe lung disease. J Air Waste Manag Assoc.

[b24-ehp-117-361] Madsen C, Nafstad P (2006). Associations between environmental exposure and blood pressure among participants in the Oslo Health Study (HUBRO). Eur J Epidemiol.

[b25-ehp-117-361] Mar TF, Koenig JQ, Jansen K, Sullivan J, Kaufman J, Trenga CA (2005). Fine particulate air pollution and cardiorespiratory effects in the elderly. Epidemiology.

[b26-ehp-117-361] O’Neill MS, Veves A, Zanobetti A, Sarnat JA, Gold DR, Economides PA (2005). Diabetes enhances vulnerability to particulate air pollution- associated impairment in vascular reactivity and endothelial function. Circulation.

[b27-ehp-117-361] Peretz A, Sullivan JH, Leotta DF, Trenga CA, Sands FN, Allen J (2008). Diesel exhaust inhalation elicits acute vaso-constriction *in vivo*. Environ Health Perspect.

[b28-ehp-117-361] Rooke GA, Feigl EO (1982). Work as a correlate of canine left ventricular oxygen consumption, and the problem of catecholamine oxygen wasting. Circ Res.

[b29-ehp-117-361] Schwartz PJ, Vanoli E, Stramba-Badiale M, De Ferrari GM, Billman GE, Foreman RD (1988). Autonomic mechanisms and sudden death. New insights from analysis of baroreceptor reflexes in conscious dogs with and without a myocardial infarction. Circulation.

[b30-ehp-117-361] Sioutas C, Koutrakis P, Burton RM (1995). A technique to expose animals to concentrated fine ambient aerosols. Environ Health Perspect.

[b31-ehp-117-361] Smyth HS, Sleight P, Pickering GW (1969). Reflex regulation of arterial pressure during sleep in man. A quantitative method of assessing baroreflex sensitivity. Circ Res.

[b32-ehp-117-361] Urch B, Silverman F, Corey P, Brook JR, Lukic KZ, Rajagopalan S (2005). Acute blood pressure responses in healthy adults during controlled air pollution exposures. Environ Health Perspect.

[b33-ehp-117-361] Venables W, Ripley BD (1994). Modern Applied Statistics with S-Plus.

[b34-ehp-117-361] Wellenius GA, Coull BA, Godleski JJ, Koutrakis P, Okabe K, Savage ST (2003). Inhalation of concentrated ambient air particles exacerbates myocardial ischemia in conscious dogs. Environ Health Perspect.

[b35-ehp-117-361] Zanobetti A, Canner MJ, Stone PH, Schwartz J, Sher D, Eagan-Bengston E (2004). Ambient pollution and blood pressure in cardiac rehabilitation patients. Circulation.

